# Costs and Consequences of the US Centers for Disease Control and Prevention's Recommendations for Opt-Out HIV Testing

**DOI:** 10.1371/journal.pmed.0040194

**Published:** 2007-06-12

**Authors:** David R Holtgrave

**Affiliations:** Department of Health, Behavior and Society, Johns Hopkins Bloomberg School of Public Health, Baltimore, Maryland, United States of America; Veteran's Administration, United States of America

## Abstract

**Background:**

The United States Centers for Disease Control and Prevention (CDC) recently recommended opt-out HIV testing (testing without the need for risk assessment and counseling) in all health care encounters in the US for persons 13–64 years old. However, the overall costs and consequences of these recommendations have not been estimated before. In this paper, I estimate the costs and public health impact of opt-out HIV testing relative to testing accompanied by client-centered counseling, and relative to a more targeted counseling and testing strategy.

**Methods and Findings:**

Basic methods of scenario and cost-effectiveness analysis were used, from a payer's perspective over a one-year time horizon. I found that for the same programmatic cost of US$864,207,288, targeted counseling and testing services (at a 1% HIV seropositivity rate) would be preferred to opt-out testing: targeted services would newly diagnose more HIV infections (188,170 versus 56,940), prevent more HIV infections (14,553 versus 3,644), and do so at a lower gross cost per infection averted (US$59,383 versus US$237,149). While the study is limited by uncertainty in some input parameter values, the findings were robust across a variety of assumptions about these parameter values (including the estimated HIV seropositivity rate in the targeted counseling and testing scenario).

**Conclusions:**

While opt-out testing may be able to newly diagnose over 56,000 persons living with HIV in one year, abandoning client-centered counseling has real public health consequences in terms of HIV infections that could have been averted. Further, my analyses indicate that even when HIV seropositivity rates are as low as 0.3%, targeted counseling and testing performs better than opt-out testing on several key outcome variables. These analytic findings should be kept in mind as HIV counseling and testing policies are debated in the US.

## Abstract 2

Scenario and cost-effectiveness analyses found that for the same programmatic cost, targeted counseling and testing would diagnose more people living with HIV and prevent more HIV infections than opt-out testing.

## Editors' Summary

**Background.:**

About a quarter of a million people in the United States do not realize they are infected with HIV. Because they are unaware of their infection, they don't get the medicines they need to stay healthy, and they may also be transmitting HIV, the virus that causes AIDS, to others unwittingly. How can public health professionals best reach such people to offer them an HIV test? There are a number of different schools of thought, the two most common of which are studied in this paper.

The first is that the best way to reach them is by simply offering every single patient in every health care setting an HIV test, but giving them the option to decline. This approach is known as “opt-out testing” (because everyone gets tested unless they choose to opt out); it has recently been recommended by the leading US government agency responsible for promoting the US public's health, an agency called the Centers for Disease Control and Prevention (CDC). The CDC says that there is no need for patients to give specific written permission for the HIV test to be done and that there is no need for health professionals to offer counseling of what the consequences of a positive test might mean for them before the test.

The second school of thought is that public health professionals should instead target their efforts towards those who are at increased risk of being HIV positive, such as those who inject drugs or who have had high-risk sex. Persons at risk of infection or transmission are offered counseling before the test, to assess their actual risk of HIV and to discuss what would happen in the event that the HIV test comes back positive. During counseling, people are also given advice on steps they can take to stay HIV negative if their test comes back negative, and to prevent infecting others if their test comes back positive. This approach to HIV testing is called “targeted counseling and testing.” While targeting can be done according to levels of risk behavior, counseling and testing services can also be targeted by focusing on geographic areas (e.g., cities) with high levels of HIV infection, or focusing on different types of clinics that serve persons at high risk of HIV infection and/or with little routine access to health care (such as sexually transmitted disease or drug treatment clinics, emergency rooms, or medical clinics in prison settings).

**Why Was This Study Done?:**

The researcher, David Holtgrave, wanted to know which of these two different approaches would be better at reaching people with undiagnosed HIV infection over the course of a one-year period. He also wanted to know the costs of each approach, and which might be better at curbing the spread of HIV.

**What Did the Researcher Do and Find?:**

He used two research techniques. One is called “scenario analysis,” which involves trying to forecast the consequences of several different possible scenarios. The other is called “cost-effectiveness analysis,” which involves comparing the costs and effects of two or more different courses of action.

According to Dr. Holtgrave's analysis, opt-out testing might reach 23% of those people who are currently unaware that they are HIV positive. The program might also prevent 9% of the 40,000 new HIV infections that occur each year in the US. The cost of averting one new infection would be US$237,149. In contrast, targeted counseling and testing might identify about 75% of people in the US now unaware they are living with HIV infection, and prevent about 36% of the new HIV infections. The cost of averting one new infection would be US$59,383. Even when the author changed several assumptions in his analysis (e.g., assumptions about levels of HIV infection or the effectiveness of counseling), he found that targeted counseling and testing still performed better (so the results are “robust” across a variety of such assumptions).

**What Do These Findings Mean?:**

These findings suggest that targeted counseling and testing would be better than opt-out testing for reaching people with undiagnosed HIV infection and for helping to stop the spread of the virus. Opt-out testing, says the author, might even make some people increase their risky behavior. For example, if someone is injecting drugs, is given an opt-out HIV test, but is never questioned about substance use or counseled, and gets an HIV-negative result, they could easily conclude that their drug injecting is not putting them at risk of becoming HIV positive.

However, it is important to note that this study has a major limitation in that it tried to predict what might happen in the future—it did not study the actual impact of the two different types of testing on a group of people. Studies such as this one, which try to predict the future, are always based on a number of assumptions and these assumptions may turn out not to be true. So we should always be cautious in interpreting the results of a “scenario analysis.” In addition, because of the assumptions made in this study, these results are only directly applicable to the US population and hence the implications for other countries are not clear.

**Additional Information.:**

Please access these Web sites via the online version of this summary at http://dx.doi.org/10.1371/journal.pmed.0040194

- In a related Perspective on this article, Ronald Valdiserri discusses the public health implications of the study
- The CDC has a Web site with information on national HIV testing resources
- In addition, the CDC has published its “Revised Recommendations for HIV Testing of Adults, Adolescents, and Pregnant Women in Health-Care Settings,” which lay out its proposal for opt-out testing
- The international AIDS charity AVERT has a comprehensive page on HIV testing, including information on the reasons to have a test and what the test involves
- Johns Hopkins University is host to a site that provides extensive information on HIV care and treatment
- The University of California at San Francisco maintains HIV InSite, an authoritative Web site covering topics such as HIV prevention, care, and policy

## Introduction

In September 2006, the US Centers for Disease Control and Prevention (CDC) recommended that routine opt-out HIV testing (testing without the need for risk assessment and counseling) be offered in all health care settings in the nation (including substance use treatment settings, correctional health care facilities, emergency and urgent care clinics, primary care settings, and all public health and community health care clinics) [1]. Such testing is to be offered to all 13- to 64-year-old persons in health care. Routine opt-out testing may eventually be discontinued in locales where HIV seroprevalence is documented at less than 0.1%. CDC acknowledged that some time-consuming aspects of HIV counseling and testing would need to be removed in order to make routine testing a reality in busy clinical settings. Consent to HIV testing would now accrue from general medical consent, not from specific written consent for the HIV test itself. No risk assessment or pretest counseling would be required. Post-test counseling may be offered for HIV-seronegative persons at high behavioral risk so long as the counseling does not become a barrier to routine testing. For persons who test HIV seropositive, linkages are to be provided to care, treatment, and prevention services, but scant detail is provided by CDC as to how this is to be achieved.

These recommendations have been met with strong reactions of both support and concern. For instance, the American Medical Association (AMA) welcomed the CDC recommendations in a press statement [2]. The AMA noted the importance of early diagnosis for persons living with HIV (especially for the roughly 250,000 persons living with HIV who do not know it), and the need to reduce HIV transmission to others. On the other hand, the American Civil Liberties Union has raised concerns about the lack of specific informed consent and the potential for coercive testing practices [3]. The National Association of People with AIDS has endorsed the expansion of HIV testing, but is concerned about informed consent issues as well as the lack of counseling services [4]. The latter is a critical issue because client-centered counseling accompanying HIV testing has been shown in a CDC-sponsored randomized controlled trial to reduce incident sexually transmitted disease infections by about 20%, and up to 40% in adolescents [5,6]. Further, when this trial was published, CDC stated that client-centered HIV counseling was both effective and practical in real-world settings [7].

Prior to CDC's recent release of opt-out HIV testing recommendations, the US Preventive Services Task Force, a panel of health care experts that evaluates the scientific evidence on clinical preventive services, chose not to recommend for or against routine testing [8]. While the task force endorsed the benefits of HIV treatment for persons testing HIV seropositive, it raised screening-related concerns about stigma, violence, abandonment, psychological impacts, and negative test results possibly providing “false reassurance if high-risk behaviors are continued” [8].

Given the level of intense discussion about the CDC recommendations, it is worth noting that neither CDC nor any of the organizations endorsing the recommendations have estimated the potential costs and consequences of routine opt-out testing. CDC has asserted that it is too early to know what the costs and consequences might be [9,10]. However, this seems at odds with the emphasis by the US Office of Management and Budget, the White House office responsible for devising and submitting the US president's annual budget proposal to Congress, on conducting cost–benefit and cost-effectiveness analyses for federal policies and programs [11]. Recent articles have used cost-effectiveness analysis to determine the cost per quality-adjusted life year saved for routine screening at various levels of background HIV seroprevalence; however, none of these publications examined the overall costs and public health impact of CDC's recommendations [12–14]. Even though neither CDC nor AMA knows the overall costs and consequences of these recommendations, both have already raised the issue of addressing state laws that require HIV-specific informed consent for testing and client-centered counseling. AMA has asked states to “re-examine” their laws so as to allow CDC's new guidelines to be carried out [2]. CDC has obliquely encouraged its partners to consider taking steps to resolve existing conflicts between the recommendations and state laws [1].

In this paper, I estimate the overall costs of CDC's routine HIV testing recommendations, estimate the potential impact of these recommendations in terms of persons newly learning of their HIV infection, and estimate the number of potential infections averted in the US (there are roughly 40,000 new HIV infections each year in the nation) [15]. Further, I estimate the medical care resources that will need to be made available quickly to meet the clinical needs of persons newly diagnosed with HIV. For comparative purposes, four distinct scenarios are examined: (a) opt-out HIV testing as recommended by CDC (the basic case analysis); (b) opt-out HIV testing that induces increases in risk behavior due to lack of counseling; (c) HIV testing accompanied by client-centered counseling; and (d) using the same level of resources needed for routine opt-out testing (as estimated in the analyses shown here), a program of HIV counseling and testing targeted to settings with a higher HIV seropositivity than is found in typical health care settings.

## Methods

Standard methods of scenario and cost-effectiveness analysis were used to analyze the costs and consequences of the four cases listed above. The analyses employed a payer's perspective [16,17] rather than a societal perspective so as to best estimate the resources needed to implement CDC's recommendations. All costs are expressed in 2005 US dollars, and all analyses were done in Microsoft Excel 2003 (http://office.microsoft.com); see Table S1 under Supporting Information for the spreadsheet containing all formulae used here. A one-year time horizon was employed so as to examine intensively the initial impact of CDC's recommendations and the alternative scenarios. (In the Discussion section, the results are interpreted for the reader interested in a societal perspective and a multiyear time horizon.)

Table 1 contains the input parameter values for the basic analysis, as well as the sources of the parameter values [18–38]. Of course, some parameter estimates contain uncertainty, and where parameter estimation called for judgment to be made, I biased the estimates in favor of CDC's opt-out testing recommendations (as described below). The parameter values related to population size are self-explanatory.

**Table 1:**
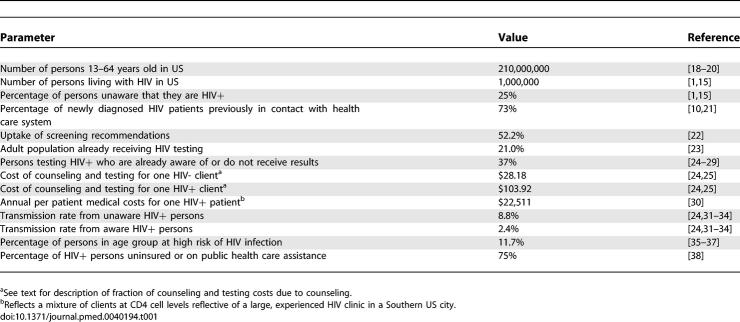
Input Parameters, Values, and Sources

CDC recently published data that show that 73% of persons diagnosed with HIV in South Carolina had previously visited a health care facility and could have been tested for HIV, had routine testing been available [10,21]. This value may be high for some populations heavily impacted by HIV with little access to health care (such as homeless youth), but is the best available estimate in the literature and likely biases the results in favor of opt-out testing.

For simplicity, it was assumed here that all incident and prevalent HIV infections in the US are among 13- to 64-year-olds; this too gives a slight bias in favor of CDC's recommendations. The analyses assume that the CDC routine HIV testing recommendations will achieve a first-year uptake of 52.2% (for a total of 109,620,000 HIV tests). This percentage is the uptake level equal to that of other recommended screening tests in the US [22]. However, 21.0% of the adult population is already being tested for HIV [23], so the actual uptake of testing due to the new recommendations is the difference between those two percentages (i.e., 31.2%).

The analyses assume that a two-step rapid testing strategy is to be used (an initial rapid HIV testing, followed by a confirmatory Western blot as necessary), as this would maximize client receipt of test results and is consistent with CDC's emphasis on using rapid testing where possible [1,25]. The full cost of delivering a testing strategy with pre- and post-test counseling (from a payer's perspective) has been estimated at US$28.18 for HIV-seronegative people and US$103.92 for HIV-seropositive people [24,25]. Prior analyses have estimated that about 61.5% of the cost of counseling and testing for HIV-seronegative clients is attributable to counseling (roughly half of that for pretest counseling, half for post-test counseling) [26], bringing the testing-only cost down to US$13.01. Removing pretest counseling for people testing HIV seropositive reduces that expense by about US$7.59 to US$96.93 (this assumes CDC would still recommend post-test counseling to HIV-seropositive persons) [24,26]. My estimates of the costs of testing services are within the ranges published by Phillips and Fernyak [39]. (It is recognized that other testing technologies are available—such as non-rapid testing options—that may have slightly different cost levels; however, this analytic framework can be used to examine any testing technology of interest to the reader.)

The analyses separately calculate the number of persons who newly learn that they are HIV seropositive, and the number of persons who already know that they are HIV seropositive but are tested (and test seropositive) again due to new testing initiatives. Prior analyses have estimated that of persons testing HIV seropositive, about 37% already know they are HIV seropositive or do not return for results [24–29]. For persons who newly learn that they are HIV seropositive, their medical care costs are estimated to be similar to those experienced by persons receiving care at a large HIV clinic in the southeastern US (where the median CD4 cell count is 367 and the range is 2 to 2,671) [30]. However, this estimate of medical need for a newly diagnosed HIV-seropositive client can be multiplied by a constant to reflect higher or lower medical costs in any clinic population of interest.

To estimate the number of HIV infections averted, I first examined transmissions prevented from persons who newly learn that they are HIV positive due to the proposed program. It has been estimated that persons who are unaware that they are living with HIV transmit at an 8.8% annual rate; persons who are aware that they are living with HIV infection have been estimated to transmit at a 2.4% rate [24,31–34]. Therefore, as persons learn of their HIV seropositivity, it is assumed that their transmission rate drops accordingly. It should be noted that omitting pretest counseling for persons who test HIV seropositive may diminish this change in transmission rate, but for the sake of simplicity such a “penalty” is not included here.

With these input parameters, I was able to calculate the following outputs using basic algebra: (a) number of persons tested under the recommended program; (b) number of undiagnosed HIV-seropositive persons newly reached; (c) total cost of testing program; (d) HIV transmissions averted; (e) gross cost per transmission averted; and (f) public sector medical care resources needed in one year to care for persons newly diagnosed with HIV infection. The analysis that calculates these outputs using the inputs described above is labeled the “Basic Case Analysis (Opt-Out Testing).”

In line with CDC's recommendations, the Basic Case Analysis (Opt-Out Testing) makes a simplifying assumption that the removal of HIV counseling for seronegative persons at high behavioral risk of infection does no harm. This assumption is counter to the literature, which notes that client-centered counseling accompanying testing can reduce incident sexually transmitted infections by 20% (or even more among adolescents) [5–7]. Therefore, I created and assessed alternative scenarios.

The “Behavioral Offset Case Analysis” is exactly the same as the Basic Case Analysis (Opt-Out Testing) with one exception. It has been estimated by CDC's National Center for Health Statistics that roughly 11.7% (11.9% in one publication) of the US population 15–44 years old is at high behavioral risk of HIV infection [35–37]. I make a simplifying assumption that this percentage holds for 13- to 64-year-olds but recognize that the actual percentage is not known and may vary by age. (Note that the Basic Case Analysis is actually the same as a Behavioral Offset Case sensitivity analysis but assumes that 0.0% rather than 11.7% of persons are at high behavioral risk.) It is possible that persons at high risk of HIV infection who are tested via CDC's recommended program (which omits risk assessment and counseling for HIV seronegative persons) could actually increase their risk behavior. For instance, if an injecting drug user who is given an opt-out HIV test without being questioned about substance use or counseled about risk gets an HIV-negative result, the individual could easily take that testing experience as a confirmation that injecting drugs is not posing an HIV-related risk. Indeed, some persons repeatedly seeking HIV testing use the experience as a risk confirmation strategy [40,41]. Further, CDC and Kaiser Family Foundation have estimated that roughly four in ten persons in the US have some basic misconceptions about HIV [42,43]. Hence, perfect HIV-related knowledge cannot be assumed among patient populations. In the Behavioral Offset Case, the rate of HIV incidence is calculated for persons at high risk of infection tested under CDC's recommended program, and it is assumed that the rate increases by 5% due to behavioral offset. This behavioral offset parameter is not known with much precision and suggests an important area for additional research.

Next, I created a Routine Counseling and Testing Case. This case assumed that clients received counseling and testing (but if someone reported no risk behaviors and tested HIV negative, no post-test counseling would be needed). While the additional counseling in this scenario would of necessity increase the cost relative to the basic program, this counseling would prevent infections among high-risk seronegative persons. The best available estimate in the literature for the effect size of counseling among high-risk seronegative persons is 20% [5–7]. So as not to overstate the case for counseling (and so as to ensure that any potential bias is in favor of opt-out testing rather than against it), that effect size is reduced here to 15%. In other words, it was assumed that the number of incident HIV infections among HIV-seronegative persons at high risk of infection is reduced by 15% due to the provision of client-centered counseling and testing. Given that a small number of HIV-seronegative persons would fail to get their results even in a rapid testing situation, this benefit is decreased by 1% (consistent with prior peer-reviewed analyses) [24].

Once the estimated cost of the Basic Case program is known, another type of analysis is possible. Recently, the US president proposed US$93 million for targeted HIV counseling and testing efforts in the US (focusing particularly on incarcerated populations, persons in drug treatment, and other persons in clinical and community-based service delivery settings at high risk of undiagnosed or imminent HIV infection). I have previously estimated that the president's proposed US$93 million counseling and testing initiative might (in one year) identify as many as 26,984 undiagnosed persons living with HIV, and prevent up to 2,537 transmissions and infections at an average cost of US$36,663 each [24]. I used this framework of a targeted counseling and testing program analysis here to ask, “At the level of resources needed to fund CDC's recommended routine testing program, what would be the impact of a targeted counseling and testing program?” Hence, a scenario labeled “Targeted Counseling and Testing” was created.

In the Targeted scenario, it was assumed that the level of available resources for service delivery was the same as that estimated in the Basic Case Analysis described above (in other words, the resource level output of the Basic Case Analysis was used as the resource level input in the Targeted Counseling and Testing analysis). Besides the other input parameters in Table 1, additional assumptions were needed for this scenario. Given that this case assumes a highly targeted program, it would be possible to essentially ensure that counseling and testing was offered only to persons at high risk of infection. However, this percentage was instead assumed to be 50%, so as to allow for some offering of counseling and testing to lower-risk persons in areas of higher HIV seroprevalence. This scenario analysis assumes that 1% of persons being tested are HIV seropositive (which is less than the 1.5% seropositivity typically seen in publicly funded HIV counseling and testing sites in the US and used in previously published analyses) [24,25]. Since this targeting might be achieved via ongoing surveillance and evaluation activities, no additional costs were allocated for additional targeting efforts or outreach; however, the assumptions about the ability of this program to reach populations with high HIV seropositivity was examined in sensitivity analyses.

## Results

Table 2 displays the results of the Basic Case Analysis (Opt-Out Testing), the Behavioral Offset Case Analysis, the Routine Counseling and Testing Case Analysis, and the Targeted Counseling and Testing Case Analysis. (Table S1 provides supplementary information on the spreadsheet used to conduct the analyses.) In the Basic Case, CDC's recommended program of opt-out testing could be expected to test 65.5 million persons, newly identify HIV infection in 56,940 out of a total of 250,000 persons in the US unaware that they are living with HIV, and avert 3,644 (out of 40,000) HIV transmissions in one year at a programmatic cost of US$864.2 million (compared to CDC's annual HIV prevention budget of a bit over US$700 million per year [44]). The cost per HIV infection averted is US$237,149. In one year, public support in the amount of US$961.3 million would be needed to provide care for persons newly diagnosed with HIV who are on public assistance or uninsured.

**Table 2:**
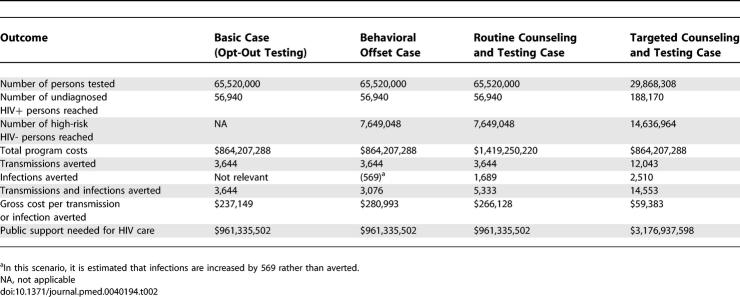
Costs and Consequences of Four HIV Testing or Counseling and Testing Scenarios

The Behavioral Offset Case Analysis found that the lack of risk assessment and counseling for the 7.6 million persons at high behavioral risk of infection tested in this program might increase infections by 569 over one year, thereby making the net number of transmissions averted 3,076 over one year (and increasing the cost per infection averted to US$280,993).

The Routine Counseling and Testing Case Analysis found that with the provision of the additional counseling services to HIV-seronegative persons at behavioral risk of infection, the cost of the program would rise to US$1,419.3 million. However, it would avert an additional 1,689 HIV infections and the cost per infection averted would be US$266,128. This cost per infection averted is intermediate between (but highly similar to both) the Basic Case and the Behavioral Offset Case.

The Targeted Counseling and Testing Case outperforms the other scenarios. It is tied for least expensive, newly identifies more persons living with HIV (188,170), prevents more transmissions (12,043), and prevents more HIV infections among at-risk seronegative persons (2,510) for a cost per infection averted of US$59,383. As this scenario outperforms the others by a wide margin, it would still perform better even if substantial additional costs were needed for targeting and outreach program components. Of course, by identifying more persons living with HIV, this scenario increases the public resources needed for medical care in one year to US$3,176.9 million.

Table 3 displays a sensitivity analysis that shows the robustness of the benefits of Targeted Counseling and Testing. Even if HIV seropositivity was as low as 0.3% and even if there was no benefit at all to providing counseling to high-risk HIV-seronegative persons, Targeted Counseling and Testing would still be (slightly) preferred over opt-out testing without counseling.

**Table 3:**
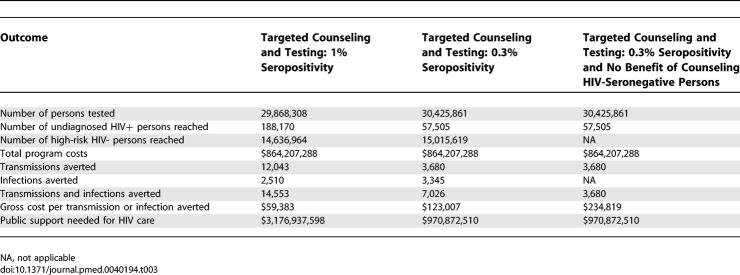
Costs and Consequences of Targeted HIV Counseling and Testing at Varying Levels of Seropositivity

## Discussion

These analyses estimate that CDC's recommended program of routine opt-out HIV testing might reach 22.8% of the 250,000 persons in the US now unaware that they are living with HIV. The program might prevent 9.1% of the 40,000 HIV infections that now occur annually in the US; if there is behavioral offset (i.e., increases in risk behavior due to lack of counseling), this percentage drops to 7.7%. Routine counseling and testing would cost substantially more to implement than opt-out testing, but would avert 13.3% of incident HIV infections in the US; considering both the additional costs and benefits, it is estimated that the cost per infection averted is roughly the same when comparing routine counseling and testing to either “testing-only” strategy.

If US$864.2 million could be made available for a testing or counseling and testing program, it would appear that the better investment would be a highly targeted program. This targeting strategy could identify roughly three-fourths of persons in the US now unaware that they are living with HIV infection, and prevent about 36.4% of the incident HIV infections. This targeted program could combine a mixture of clinical and community-based counseling and testing services to achieve a 1% seropositivity rate (and therefore, may represent a hybrid clinical/community-based strategy rather than a strictly community-based approach). This finding is robust to changes in HIV seropositivity levels and effectiveness of client-centered counseling.

Of course, there are multiple limitations to the current analysis. Many of the parameters in Table 1 are subject to uncertainty (as acknowledged above). My analyses must be continually updated as better parameter values become available and policy descriptions are sharpened (indeed, CDC has stated it will issue supplemental implementation guidance over time) [10]. Further, I focused on the first-year costs and consequences of the recommendations so as to illuminate the immediate ramifications of the recommendations. In future research, a multiyear analysis would be desirable. However, the patient population, the health care sector, the media, legislative bodies, and society at large are always changing, and their reaction to the rollout of any of the scenarios described above is impossible to predict with complete precision; this changeability will make any multiyear analysis an ongoing project. Nevertheless, the basic results of a multiyear analysis can be anticipated. Holtgrave and Pinkerton recently estimated the number of new HIV infections per year over a ten-year time horizon in the US at various levels of awareness of HIV seropositivity (from 75% awareness to 100%) [34]. They found a divergent pattern in incidence over time given various starting levels of awareness of HIV seropositivity. For example, using an HIV-specific death rate to reflect mortality, at 75% awareness, annual HIV incidence would grow from 40,000 to 48,526 over a ten-year time frame. However, if 95% awareness of seropositivity were achieved in one year, annual HIV incidence would start at 27,200 in one year and grow more slowly to 29,458 over a decade. If the four service delivery scenarios described in the present paper were all one-year campaigns (with all programmatic costs incurred in year one), then such a divergent pattern of HIV incidence levels in subsequent years might be anticipated; in other words, if the targeted counseling and testing scenario modeled here is to be preferred in year one in terms of reduced HIV transmission and incidence, it is also likely to be preferred in downstream years as well in terms of reduced transmission and incidence. Of course, if the scenarios used counseling and testing campaigns of relatively different time frames, then a new multiyear analysis would be required.

Also, due to space limitations not all of the sensitivity analyses performed in this study are presented here; however, given that the formulae employed are straightforward algebraic expressions, the results of these sensitivity analyses are easily anticipated (and can be confirmed with the spreadsheet in Table S1). Table 3 presents what was found to be the most important scenario analysis, which provided assurance of the superiority of a targeted testing strategy even under rather extreme parameter assumptions.

I focused here on a payer's perspective so as to highlight the resources needed to implement CDC's recommendations or any of the other scenarios. Because of the substantial diagnostic and preventive benefits of the targeted counseling and testing strategy, adding in productivity benefits due to early treatment or medical costs saved by averting more HIV infections (as necessitated in a societal perspective analysis) would only serve to make the targeted strategy appear even more favorable. For instance, in a societal perspective analysis, one could legitimately subtract from the cost per HIV infection prevented by a counseling and testing program the discounted, lifetime medical care costs of US$278,078 to US$316,149 (2005 dollars) for one case of HIV disease averted [45,46]. Given the gross costs per HIV infection averted displayed in Tables 1 and 3, such a subtraction would lead us to classify the scenarios as “cost-saving” (or very nearly so). The only countervailing force would be if one differentially added in client costs due to time spent in service delivery and travel to receive targeted services; however, in sensitivity analyses it was found that even if societal perspective client costs doubled the per-client, payer perspective costs of targeted HIV counseling and testing, the targeted strategy still very clearly outperformed the opt-out strategy. Hence, shifting to a different analytic perspective would not modify my overarching, qualitative conclusion here.

Despite their limitations, the analyses presented here are useful for several reasons. First, by laying out an explicit framework for estimating the costs and consequences of CDC's recommended opt-out testing program, the analyses help to sharpen the discussion about the merits of the recommendations. Second, the analyses highlight research needs regarding the value of various input parameters necessary to further refine that discussion. Third, even with sources of uncertainty, the analyses give a general sense of how much work will remain to be done in the HIV epidemic even with such a program in place. Indeed, none of the scenarios presented here should be mistaken for a comprehensive HIV prevention program—testing or counseling and testing are important but only a piece of a comprehensive national plan [37].

Fourth, the present analyses provide a general estimate of the prevention and treatment resources that will need to be raised to make CDC's recommendations a reality. Fifth, by estimating the costs of CDC's recommendations, this analytic framework allows for comparison of alternative counseling and testing policies at a specified resource level. On this point, the analyses presented here suggest that to maximize public health impact, targeting and counseling elements should be carefully considered for inclusion in national testing policies. Given these strengths, the current analysis provides insights beyond previous cost-effectiveness analyses of expanded HIV testing [12–14,47].

## Supporting Information

Table S1 Scenario Analysis Spreadsheet for Estimating Costs and Consequences of Four HIV Testing or Counseling and Testing Scenarios (45 KB XLS)

## Abbreviations

AMA: American Medical Association
CDC: Centers for Disease Control and Prevention
